# Supplementary material for: Foreskin surface area is not associated with sub-preputial microbiome composition or penile cytokines
Source: PLoS One. 2020 Jun 23;15(6):e0234256. doi: 10.1371/journal.pone.0234256 (PMC7310704; doi:10.1371/journal.pone.0234256)
Supplement: Attachment — Submitted filename: Response to reviewers.docx [file pone.0234256.s002.docx]

**Reviewer #1:** In this manuscript, Kigozi et al. analyzed the association between the foreskin surface area and HIV-1 risk by measuring the correlation between foreskin size and anaerobes or pro-inflammatory cytokines. While I found this work of interest, the following concerns should be raised.

1. Manuscript type. This study reports a very specific result of weak correlations between the foreskin surface area and anaerobes or pro-inflammatory cytokines. Considering the significance and the manuscript length, in this case, I would suggest the authors to format it as a “Brief report”, “Communications” or “Letter to the editor” rather than a “Research article”.

*- We apologize for any lack of clarity. The goal of our report is not to imply that there are weak correlations between foreskin surface and either microbiome or immune parameters – rather, it is to demonstrate that there is no correlation. At scientific meetings, we are frequently questioned if there is a relationship between foreskin size and anaerobe burden, and this analysis is meant to address that question. We hope that the data presented are now clear and make a consistent story. Since the mandate of Plos One is to publish valid research regardless of whether the results are positive or negative, we hope that they will be amenable to publishing these negative results in full.*

*- With regards to manuscript format, Plos One does not offer the manuscript formats suggested by the reviewer. Plos One has a relatively broad definition for Research Articles (“reports the results of original primary research, including quantitative and qualitative studies, methods and software studies, systematic reviews, and other work”), with no word count minimum or maximum.*

2. Technical issues. The analysis on abundance of bacteria was based on 16S rRNA profiling, mainly on Genus-level. I strongly recommend the authors to (a) go deeper on 97% or 99% OTU level for higher precision, and (b) also from the functional aspect using 16S-based function annotation method (e.g. PICRUSt).

*- Thank you for this suggestion. Data are shown to the 97% OTU level, as recommended.*

3. In the current results, since there is no individual genus was associated with the foreskin surface area that may influence the risk of HIV-1, is there any probability that the risk is correlated with the combination of multiple organisms? A machine-based approach / regression analysis of multiple-taxon / PCA may inspire new findings, or further confirm the current results.

*- In response to this excellent suggestion we conducted decision tree analyses to evaluate potential associations of combinations of bacterial taxa with surface area. For this analysis, we also expanded to use the full set of bacterial taxa found in the sub-preputial space, as opposed to limiting to those taxa previously associated with circumcision. Only one taxon (an OTU with 80% similarity to* Gallicola*) was identified as positively associated with surface area. Forcing an expansion of tree depth did not identify any informative or significant combinations of multiple organisms. The one taxon identified as being associated with surface area was only marginally informative (r=0.280), and although the association had a significant Spearman correlation, this taxon had a mean proportional abundance of less than 0.1% and is unlikely to be of biological relevance.*

4. Line #126, for specific anaerobic genera previously found to be associated with HIV-1 risk, each of them should be cited, or linked to the previous studies (e.g. listed in a table to summarize each of the genera and its citation(s)).

*- The relevant citation had been added.*

5. The resolution of Figure 1 should be improved.

*- This figure has been uploaded as a high-resolution tiff, and we hope that the reviewer is now able to view it in a high res format.*

**Reviewer #2:** Thank you for the opportunity to review this brief, but well-written manuscript. The study appears well-conducted and contributes novel information to the study of MMC and HIV risk. I have only minor comments.

*- Thank you for the kind comments.*

Methods:
• It seems unusual to have ethics information at the end of the methods. If this is a journal style requirement, fine, but otherwise it is more common to lead with ethics information.

*- These have now been placed at the start of the Methods, as suggested.*

• Which R version was used? Any specific packages?

- *The R version used was R Development Core Team, Version 3.3.1. This information has now been added to the Methods section (lines 116-118).*

Results:
• Graphical results of numeric IL-8 analysis are mentioned in the methods but not presented.

*- Thank you, the methods have been corrected (line 112).*

• It’s possible that the genera previously associated with HIV risk are incorrect. Was a larger analysis conducted? I don’t necessarily advocate a fishing expedition, but given the negative results, perhaps other genera might be important here.

- *Our prior publication demonstrated eight genera associated with HIV risk in uncircumcised men, but the reviewer is correct that the analysis was limited to genera that had previously demonstrated to be reduced by circumcision. To determine if other taxa are associated with surface area, we performed additional analyses expanding to use the full set of bacterial taxa found in the sub-preputial space. Only one taxon (an OTU with 80% similarity to* Gallicola*) was identified as being marginally informative in predicting surface area (positive association, r=0.280). Although the association had a significant Spearman correlation, this taxon had a mean proportional abundance of less than 0.1% and is therefore unlikely to be of biological relevance.*

Discussion:
• I am not sure about the strength of the conclusions given the fact that this is a single, relatively small study. Changing “indicating” to “suggesting” on line 155 may adequately temper the language.

- *We have tempered the wording, as suggested.*

Supplement:
• Total bacterial load and quantitative IL8 data are not present in the spreadsheet

*- Our apologies for the oversight, those data have now been added.*
