# Supplementary material for: Foreskin surface area is not associated with sub-preputial microbiome composition or penile cytokines
Source: PLoS One. 2020 Jun 23;15(6):e0234256. doi: 10.1371/journal.pone.0234256 (PMC7310704; doi:10.1371/journal.pone.0234256)
Supplement: Attachment — Submitted filename: response to comments.docx [file pone.0234256.s003.docx]

Dear Dr. Hickok

Our group has previously published two independent analyses using data collected during the randomized controlled trial of circumcision in Uganda: one showing an association between foreskin surface area and risk of HIV acquisition (*Aids* 2009), and a second analysis showing associations between penile anaerobes, pro-inflammatory cytokines, and HIV risk (*MBio* 2017). However, our group (and, to our knowledge, no other group) has previously assessed the relationship between foreskin surface area and penile anaerobes and/or pro-inflammatory cytokines. Therefore, the analyses presented in the current manuscript are not an update on any previous findings. I apologize for this lack of clarity, and have altered the wording in the introduction in lines 79-80 to try to address this issue.

Another concern raised was the possibility of selection bias for enrolment of men who had been exposed to HIV but remained uninfected. At the time of enrolment in the trial there was no evidence that circumcision reduced HIV risk, and thus enrolment was performed at a community level without selection based on sexual activity or prior HIV exposure. Additionally, the present analyses were performed on samples collected at trial initiation, and thus no additional selection bias was introduced by a requirement of remaining HIV negative throughout the trial period. This has now been clarified on lines 93-95.

I hope that we have adequately addressed your two remaining concerns, and have uploaded a new version of the manuscript with these changes tracked. Thank you for considering our manuscript for publication in *Plos One*.

Sincerely,

Jessica Prodger
